# Proline metabolism and redox; maintaining a balance in health and disease

**DOI:** 10.1007/s00726-021-03051-2

**Published:** 2021-07-22

**Authors:** Lisa A. Vettore, Rebecca L. Westbrook, Daniel A. Tennant

**Affiliations:** Institute of Metabolism and Systems Research, College of Medical and Dental Sciences, University of Birmingham, Edgbaston, Birmingham, UK

**Keywords:** Proline, Redox, Cancer, Disease

## Abstract

Proline is a non-essential amino acid with key roles in protein structure/function and maintenance of cellular redox homeostasis. It is available from dietary sources, generated de novo within cells, and released from protein structures; a noteworthy source being collagen. Its catabolism within cells can generate ATP and reactive oxygen species (ROS). Recent findings suggest that proline biosynthesis and catabolism are essential processes in disease; not only due to the role in new protein synthesis as part of pathogenic processes but also due to the impact of proline metabolism on the wider metabolic network through its significant role in redox homeostasis. This is particularly clear in cancer proliferation and metastatic outgrowth. Nevertheless, the precise identity of the drivers of cellular proline catabolism and biosynthesis, and the overall cost of maintaining appropriate balance is not currently known. In this review, we explore the major drivers of proline availability and consumption at a local and systemic level with a focus on cancer. Unraveling the main factors influencing proline metabolism in normal physiology and disease will shed light on new effective treatment strategies.

## Background

Proline is a non-essential proteinogenic amino acid that is available from both diet and endogenous synthesis. It has a unique cyclic structure—a pyrrolidine ring in which the side chain is connected to the amino group to form a secondary amine—that confers the amino acid significant rigidity. This makes proline an important structural component of proteins, providing the tight turns often required between secondary structural elements and allowing both cis- and trans- conformation in protein backbones (Chow et al. 2018; Lummis et al. 2005). Another particular role of proline is its apparent essentiality during stress situations (Hayat et al. 2012). Through its function as an osmolyte, antioxidant, signaling molecule, and even a metal chelator, it has been shown to combat stress conditions in plants (Hayat et al. 2012). However, this appears to be concentration-dependent, as while supplementation of exogenous proline was shown to increase stress tolerance, high-doses-induced toxicity in plants (Roy et al. 1993). Modulation of proline concentrations in mammalian cells has more recently been described, particularly in the context of disease. Although it is not yet clear under what conditions this occurs, concepts arising from the study of plants suggest that proline synthesis and degradation may play an essential role in response to cellular stress, as well as conventional roles in protein synthesis. It is, therefore, crucial to explore the drivers of proline uptake and metabolism and their association with disease, as this may identify opportunities to alter the pathogenesis of some human diseases, including cancer. In this review, we will discuss our current knowledge of the determinants of proline availability and consumption—both at the cellular and systemic level—with a particular focus on cancer as a disease in which this has been studied. We will also discuss the concept of proline synthesis and degradation as a metabolic hub to maintain redox homeostasis and the implications of this for systemic proline availability.

### Systemic proline metabolism

**Fig. 1:**
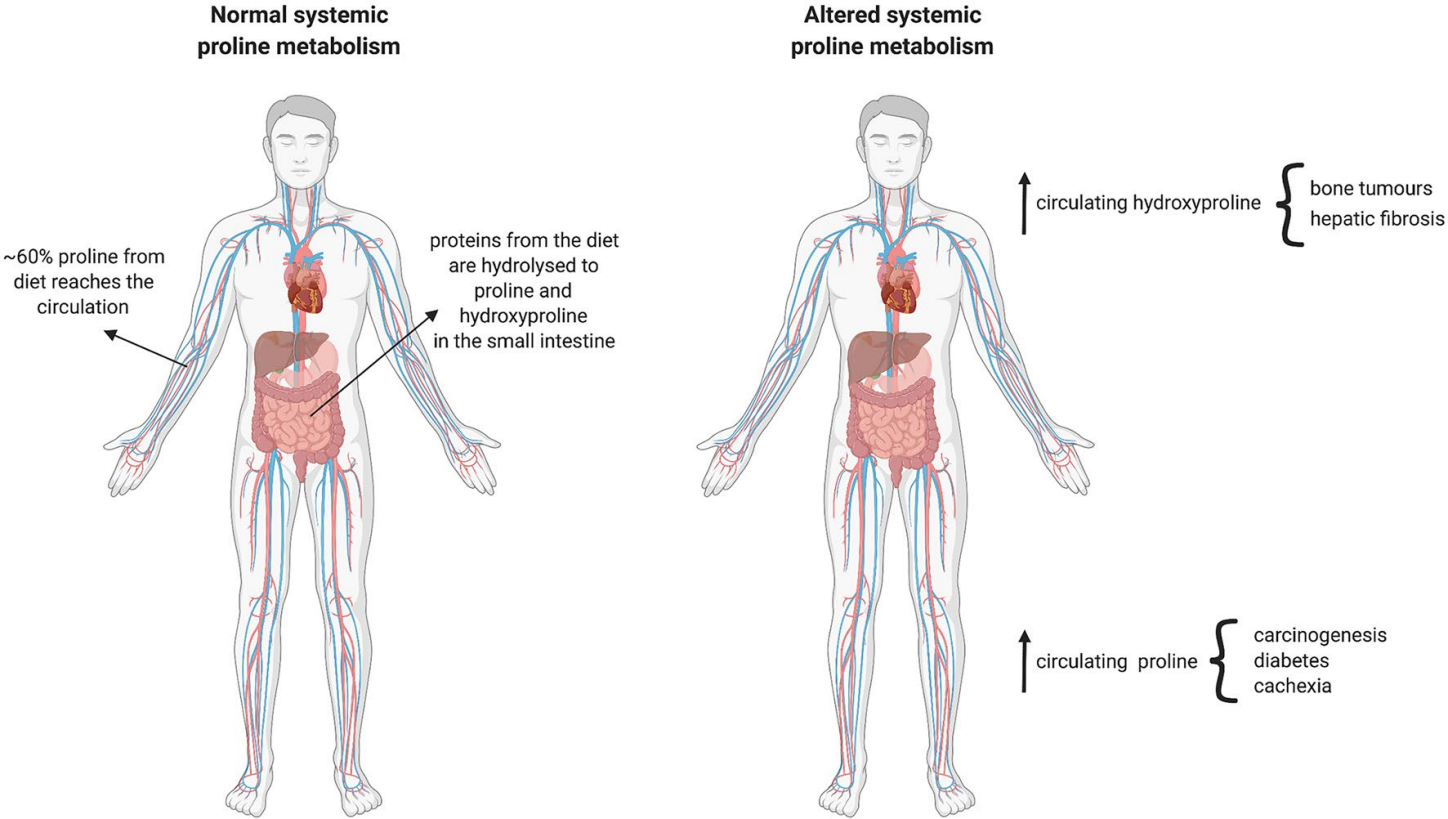
Schematic representation of systemic proline metabolism in health and disease. In normal conditions, proline and hydroxyproline are assimilated from the diet in the small intestine and about 60% will be released by enterocytes into the circulation. During disease, enhanced hydroxyproline levels are associated with bone tumours and hepatic fibrosis, whereas increased systemic proline levels mirror carcinogenesis, diabetes and cachexia

Despite the ability of mammalian cells to synthesize proline, dietary sources of proline are thought to be essential to maintain healthy function (Watanabe et al. 1999; Yam et al. 2019). In normal conditions, dietary proteins are hydrolyzed in the small intestine to release proline and hydroxyproline (Fig. 1), the latter arising from post-translational modification of prolyl residues. Of particular importance in this process are prolidases, which specifically hydrolyze prolyl or hydroxyprolyl dipeptides (Wu et al. 2011). The intestinal microbiota is thought to preferentially catabolize hydroxyproline over proline, producing acetate and propionate as a result, which can be further metabolized both locally, and by the liver (Wu et al. 2011). Absorption from the lumen is then facilitated by Na^+^-dependent IMINO transporters, the Na^+^-independent L-proline transporter, and the neutral brush-border (NBB) system, which shuttles neutral amino acids (Wu et al. 2011). While enterocytes utilize significant proportions of this proline,  ~ 60% is thought to reach the plasma (Wu et al. 2011) (Fig. 1). In proline-deficient conditions, enterocytes have been suggested to at least partially compensate, increasing their endogenous synthesis of proline. However, up to 50% of this is absorbed on first pass by the liver (Yam et al. 2019), leaving the remaining proline for uptake by other organs. Normal plasma concentrations have been reported as relatively high (100–250 μM Schmidt et al. 2016; Mc et al. 1957; Frame 1958; Martinez et al. 1993) when compared to other amino acids. Interestingly, the blood–brain barrier (BBB) restricts the entry of several amino acids, including proline (Oldendorf 1971; Sershen and Lajtha 1976; Yudilevich et al. 1972). Nevertheless, tumors themselves and brain metastasis development can disrupt BBB vasculature, resulting in the so-called brain-tumor-barrier (BTB), a leakier and permeabilized vasculature, allowing the influx/efflux of molecules that cannot access the brain in normal physiology and promotes accumulation of waste products (Arvanitis et al. 2020). This clearly has implications for the metabolic network of the central nervous system, altering the nutrient availability and requirements during disease.

Interestingly, plasma concentrations of proline and hydroxyproline are often perturbed in patients with some chronic pathologies such as diabetes and cancer, as well as through more well-described inborn errors of metabolism (Fig. 1). Increased blood hydroxyproline levels have been suggested as a biomarker of hepatic fibrosis and associated with other diseases where fibrosis is a hallmark (Gabr et al. 2016). Increased plasma proline has been observed in patients affected by type 2 diabetes, obesity, insulin resistance (Liu et al. 2016), and as a result of cancer-associated cachexia (Newton et al. 2019) (Fig. 1). In a Drosophila tumor model, it was shown that cachexic muscles released increased proline through the upregulation of two potential proline transporters, SLC36A1 and SLC36A4 (Newton et al. 2019). Interestingly, hyperprolinemia itself has also been shown to lead to metabolic consequences through amino acid toxicity and dysregulated β-cell function in INS1-E insulinoma cells and isolated mouse islets (Liu et al. 2016). Under these circumstances, insulin promoter factor 1 (Pdx1) mRNA expression was found to be increased, while the acetyl-CoA carboxylase 1 (ACC1)-AMPK axis was downregulated, indicating re-wiring of β-cell phenotype (McAnulty 2007). This dysfunction driven by chronic high levels of proline manifested as impaired insulin secretion, and therefore systemic dysregulated glucose homeostasis.

**Fig. 2:**
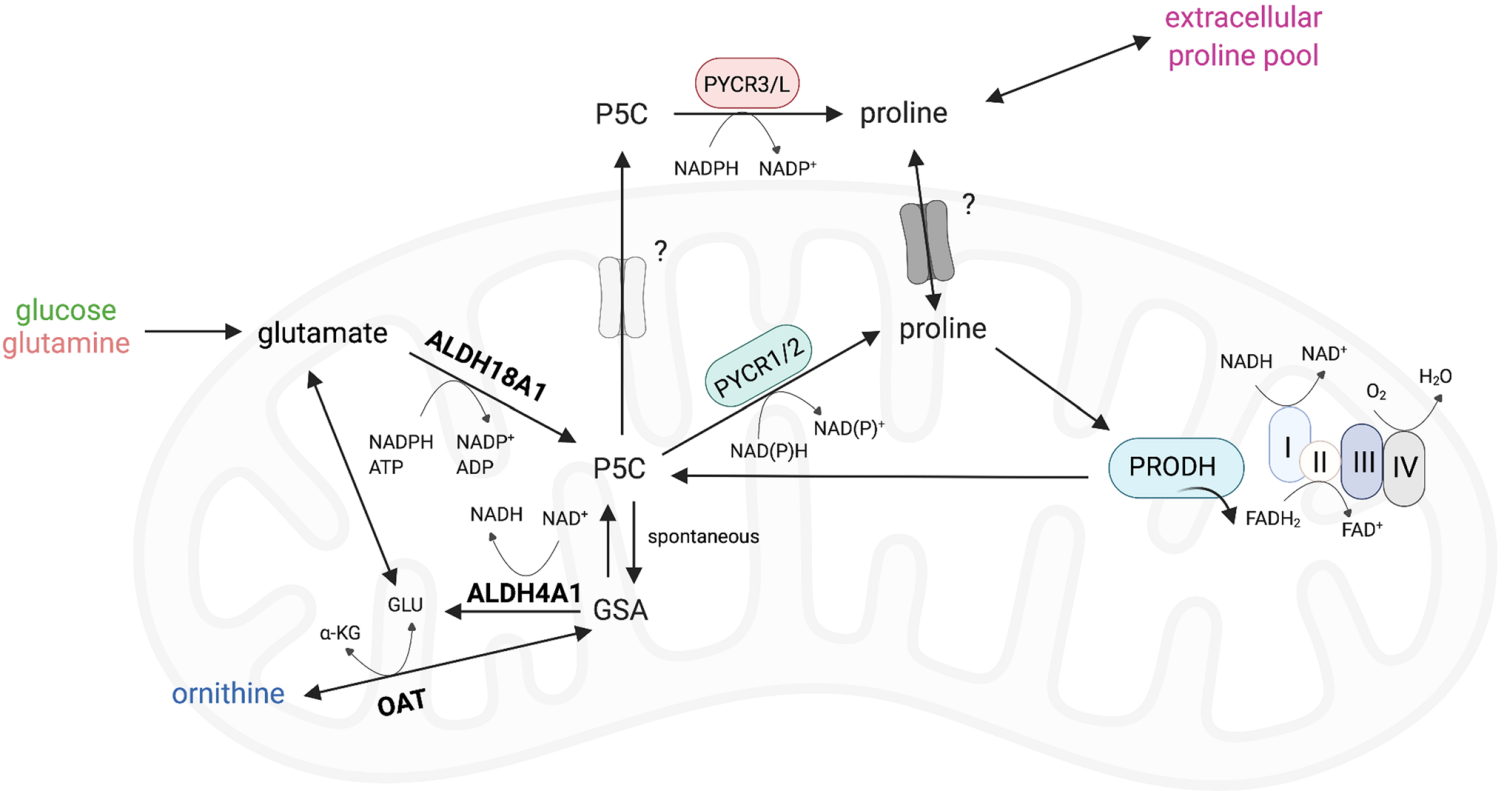
Proline metabolism at a glance. The three main carbon sources for proline biosynthesis are glucose, glutamine and ornithine, which foster the reactions of PYCR1, 2 and 3/L. Proline and extracellular proline can then be recycled by PRODH during nutrient starvation or metastasis formation. Almost all the reactions of this pathway contribute to cellular redox control. Abbreviations: α-KG; α-ketoglutarate, GLU; glutamate, P5C; 1-pyrroline-5-carboxylate, GSA; glutamate-5-semialdehyde, NAD^+^; nicotinamide adenine dinucleotide, NADH; reduced NAD^+^, NADP^+^; nicotinamide adenine dinucleotide phosphate, NADPH; reduced NADP^+^, FAD^+^; flavin adenine dinucleotide, FADH_2_; reduced FAD^+^

Another example of systemic alterations in proline homeostasis is observed in inborn errors of metabolism (Mitsubuchi et al. 2008). For instance, hyperprolinemia type I is a result of proline dehydrogenase (PRODH) deficiency, with patients presenting with velo-cardio-facial syndrome and often developing schizophrenia (Mitsubuchi et al. 2008). Hyperprolinemia type II is caused by a lack of the enzyme that contributes to the degradation of proline, ALDH4A1 (Fig. 2), and is associated with significant urea cycle dysfunction, manifesting in hyperammonemia, hypoornithinemia, hypocitrullinemia and hypoargininemia (Mitsubuchi et al. 2008).

Although dietary proline contributes significantly to proline availability, endogenous sources can contribute considerably (and sometimes pathogenically) to the overall proline pool. The mechanisms that maintain local proline levels—whether protein (particularly collagen) degradation or enhanced synthesis—are not entirely clear.

### Local proline metabolism: synthesis

Local proline concentrations are the result of a balance struck between availability from plasma, local use/degradation and local synthesis/release. The latter is the result of a balance between endogenous synthesis and the result of proteolysis—in particular, the proline-rich structural protein, collagen. When proline availability or requirements change—perhaps as the result of the anabolic drive that characterizes cancer or a change in exogenous availability due to perturbations in vascular flow—the compensatory changes required in this fine balance can significantly alter the wider cellular metabolic network.

Proline supply/demand and the wider metabolic network are significantly linked as proline synthesis and degradation are both highly redox-active processes. Proline synthesis, whether from glucose, glutamine or ornithine, is coupled to oxidation of reduced pyridine nucleotides (NAD[P]H) through the activity of Aldehyde dehydrogenase 18 family member A1 (ALDH18A1) and/or Pyrroline-5-carboxylate reductase 1,2 or 3/L (PYCR1, 2 or 3/L) (Phang et al. 2012) (Fig. 2). Conversely, proline degradation involves the activity of Proline dehydrogenase 1 (PRODH) and Aldehyde dehydrogenase 4 family member A1 (ALDH4A1), the former donating electrons to FAD^+^, while the latter reduces NAD^+^ (Phang 2019). It is therefore clear that proline and redox homeostasis are intrinsically linked and part of a critical network for the maintenance of healthy cells. Conversely, when redox homeostasis is challenged, as observed in many diseases, this is likely to have implications for endogenous proline synthesis and catabolism.

There is a significant and growing body of evidence showing that proline is used as a means of maintaining a redox homeostasis that permits normal cell function—both in cancers and in conditions associated with inflammation and fibrosis (Hollinshead et al. 2018; Schworer et al. 2020). Intriguingly, while two of the biosynthetic enzymes, PYCR1 and 2 are mitochondrially-localized, PYCR3/L is cytosolic (De Ingeniis et al. 2012) (Fig. 2). This could suggest that they have contrasting roles, responding to perturbations in redox homeostasis in different compartments. This is particularly clear in patients lacking PYCR1 or 2, as well as models of PYCR1 and 2 deficiency, which display phenotypes linked with dysregulated redox control (oxidative stress and enhanced apoptosis) (Nakayama et al. 2015; Reversade et al. 2009). PYCR1 activity has been more directly shown to oxidize mitochondrial NADH, effectively uncoupling the reducing activity of the TCA cycle from the oxidizing activity of the electron transport chain (ETC), and therefore respiration (Hollinshead et al. 2018). This metabolic pathway can therefore act as a means of allowing continued TCA cycle activity while reducing oxygen consumption—permitting the synthesis of macromolecular precursors (for new DNA/RNA and proteins) when the mitochondrial NADH:NAD^+^ ratio is high. This type of metabolic compensation to correct an unfavorable NADH:NAD^+^ ratio is well-described in the cytosol, where a high NADH:NAD^+^ balance will drive the enhanced reduction of pyruvate to form lactate that is then excreted from the cell (Tennant et al. 2009). However, there is no well-defined mechanism for ‘dumping’ excess reducing potential within the mitochondrial matrix other than using the malate-aspartate shuttle to move it to the cytosol. Indeed, in the matrix, tight coupling of TCA cycle activity to respiration is assumed as it maximizes ATP generation through the oxidation of carbon sources. However, the activity of PYCRs in the maintenance of redox homeostasis appears to be an additional mechanism, as knockdown of any of the PYCR enzymes has been shown to result in reduced intracellular lactate (Liu et al. 2015). Interestingly, there is recent evidence that some tumors import and metabolize lactate to fuel the TCA cycle, which would increase the reduction of cytosolic NAD^+^ (Faubert et al. 2017). The implications of this metabolic activity on proline metabolism have not as yet been reported.

In hypoxic conditions, the stabilization of the oxygen sensor HIF-1α through the inhibition of prolyl hydroxylases (PHDs) has been shown to increase the secretion of free proline into the microenvironment in TGF-β-induced fibroblasts (Schworer et al. 2020). This increase was shown to be reversed with the addition of alpha-ketobutyrate, which acts to normalize the NADH:NAD^+^ ratio. Knockdown of ALDH18A1 to limit proline synthesis and pharmacological inhibition of the ETC significantly reduced cell proliferation in hypoxia (Liu et al. 2020a). Interestingly, knockdown of ALDH18A1 in a xenograft model also sensitized tumors to treatment targeting fatty acid synthesis, a highly redox-active synthetic pathway (Liu et al. 2020a).

**Fig. 3:**
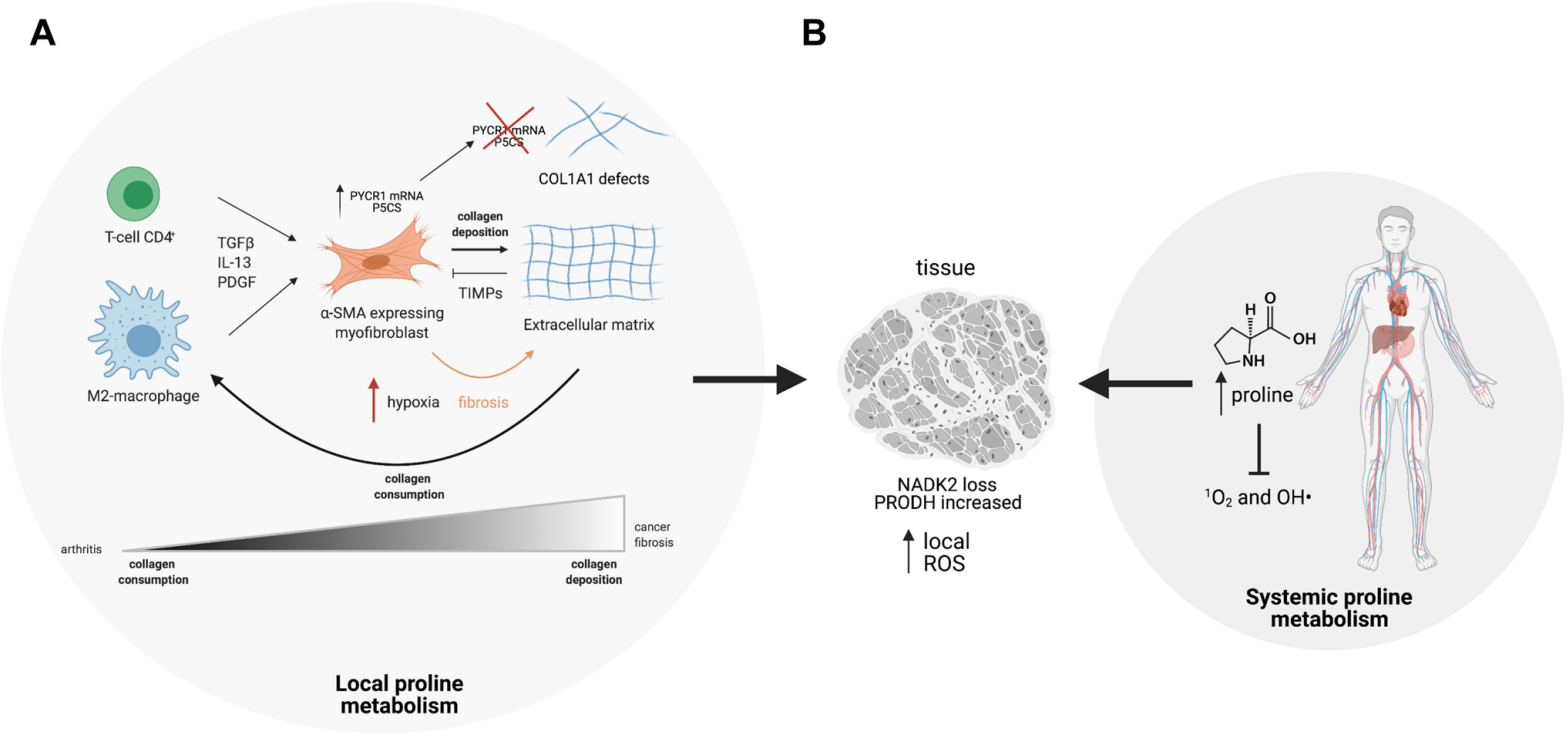
Overview of the interactions between local and systemic proline metabolism. **A** Locally M2-macrophages and T CD4^+^ cells release factors capable of transforming fibroblasts into myofibroblasts. In this condition, activated fibroblasts secrete collagen which, in turn, can be consumed by macrophages. Chronic changes of the equilibrium between collagen synthesis and deposition, cause disease. **B** Local dysfunctions in proline turnover can be compensated by systemic proline metabolism. *TGFβ* transforming growth factor beta, *IL*-*13* Interleukin-13, *PDGF* platelet-derived growth factor, *TIMPs* tissue inhibitors of metalloproteinases, ROS reactive oxygen species

While enhanced proline synthesis under these conditions is likely a phenotype to maintain intracellular redox, one cannot ignore the result of increased extracellular proline supply that arises as a consequence. Two chronic pathologies in which this redox-mediated increase in local proline concentrations might play a role are chronic inflammation and cancer, both of which are associated with significant hypoxia. During inflammation, inflammatory cells (M2-polarized macrophages and T helper 2 (Th2 cells) release cytokines that drive activation of myofibroblasts (Wynn 2008) (Fig. 3A). Amongst other phenotypes, this subgroup of fibroblasts is specialized in ECM deposition, including the synthesis of collagen, 30% of which can be prolyl residues (Wynn 2008). While this is an acute process during normal wound healing, chronic inflammation can drive longer-term fibrosis and tissue remodeling (McAnulty 2007). Fibrosis involves the chronic deposition of collagen in the local tissue, increasing ECM stiffness, leading to maladapted tissue architecture (Distler et al. 2019). This increased collagen synthesis requires significant amounts of proline, which could be derived from local sources as previously suggested (Schworer et al. 2020). Another disease that can be characterized by chronic inflammation and often significant hypoxia is cancer (the ‘wound that cannot heal’ paradigm). Interestingly, overexpression of PYCR1 has been shown in many cancer types and has even been suggested to be directly pro-tumorigenic (Burke et al. 2020). ALDH18A1 protein levels and PYCR1 mRNA have also been shown as upregulated in cancer-associated fibroblasts (CAFs), and silencing of these enzymes perturbed collagen formation, in particular collagen type I, α1 (COL1A1)—a crucial extracellular matrix (ECM) component (Kay et al. 2020) (Fig. 3A).

Interestingly, it has also been reported that hypoxia-mediated HIF-1α stabilization plays a role in collagen deposition via pyruvate-driven α-ketoglutarate (α-KG) production, which, in turn, increased collagen hydroxylation (Stegen et al. 2019). This process, observed in chondrocytes, resulted in increased ossification as the collagen hydroxylation drove a more protease-resistant matrix (Stegen et al. 2019). During chronic hypoxia, increased collagen secretion has been observed in hepatic stellate and renal cells. In fact, HIF-1α stabilization promotes the transition from fibroblasts to myofibroblasts (Corpechot et al. 2002; Orphanides et al. 1997). For this reason, a dysregulated HIF-1α signal may result in enhanced matrix stiffness, a condition which, in turn, primes fibrosis and cancer (Stegen et al. 2019). Interestingly, the metabolic support for hydroxylation of prolyl residues and synthesis of a stiffer collagen network may be essential in metastasis formation, as it was recently shown that pyruvate is required to drive this process in breast cancer (Elia et al. 2019). In agreement with this, prolyl 4-hydroxylase alpha 2 (P4HA2), which catalyzes the generation of 4-hydroxyproline, has been shown to increase matrix stiffness in lung cancer (Pankova et al. 2019).

An alternative means by which proline availability may directly alter redox homeostasis is due to the fact that proline itself has been shown to scavenge ROS (O_2_^−^ and OH^•^) (Kaul et al. 2008; Phang et al. 2012; Smirnoff and Cumbes 1989) (Fig. 3B). A recent study suggested that proline metabolism was changed after myocardial infarction, but importantly that proline supplementation was able to reduce oxidative stress and apoptosis (Wang et al. 2020). Indeed, this phenotype appears to be through more widespread transcriptional changes, as proline supplementation was able to increase Glutathione peroxidase 1 (Gpx1) mRNA levels and downregulate Thioredoxin-interacting protein (Txnip)—the inhibitor of the antioxidant system thioredoxin, further enhancing the ability of myocytes to resist oxidative stress (Wang et al. 2020).

Proline availability not only balances redox homeostasis but also influences proteostasis. A previous study has suggested that proline restriction limits proline-tRNA availability, thus impairing protein synthesis (Loayza-Puch et al. 2016). As a result of proline shortage, PYCR1 was upregulated to compensate, and in an in vivo model, PYCR1 KO halted tumorigenic growth (Loayza-Puch et al. 2016). The impact of proline on protein synthesis has also been confirmed in another study, where mitochondrial NAD kinase 2 (NADK2) provides NADP^+^, the co-factor essential to sustain proline biosynthesis through ALDH18A1. Lack of proline impaired cell growth by affecting both protein synthesis as well as purine and pyrimidine synthesis. Supplementation of proline was able to rescue the defect caused by the loss of NADK2 (Diehl and Vander Heiden 2021; Zhu et al. 2021). Taken together, these data suggest that not only protein synthesis but also nucleotide homeostasis is influenced by proline levels.

Integration of systemic and local proline availability, therefore, influences the cellular NAD(P)^+^/NAD(P)H, protein and nucleotide balance, and in doing so, the ability of cells to maintain a healthy metabolic network. In turn, perturbations in redox, protein, and nucleotide homeostasis within one cell (or cell type) can directly alter local proline availability, thereby potentially influencing the state of other nearby cells and further shaping the tissue microenvironment.

### Local proline metabolism: catabolism

Some cell types are more prone to consume extracellular proline. For instance, normal retinal pigment epithelial cells consume large amounts of free proline compared to other tissues, and export proline-derived TCA cycle intermediates which are taken up by local photoreceptor cells. This interlinked proline metabolic pathway forms a community metabolic network that benefits both cell types (Chao et al. 2017; Yam et al. 2019). On the other hand, M2-like macrophages have been suggested to be a major consumer of collagen, although the drivers of this process are not yet well-understood (Madsen et al. 2013). An imbalance of the collagen cycle—the sum of collagen producers and consumers—towards increased ECM degradation has often been observed during disease, such as arthritis (Fig. 3A). In this autoimmune disease, type II collagen is attacked by leukocytes, with monocytes/macrophages seemingly central in the progression of the disease (Guo et al. 2018). The ability to digest the ECM is also crucial for cancer invasion and metastasis. Matrix metalloproteinases (MMPs), enzymes involved in the catabolism of ECM are often overexpressed in malignant growths (Kessenbrock et al. 2010). Along with collagen degradation, the proline-consuming enzyme PRODH, also known as proline oxidase, has been shown to be upregulated in breast cancer-derived metastasis (Elia et al. 2017). Interestingly, PRODH was also shown as capable of promoting tumor progression in non-small cell lung cancer (NSCLC) as part of a network involving a chromatin remodeler, the lymphoid-specific helicase (LSH) (Liu et al. 2020b).

PRODH passes electrons from proline to FADH_2_ forming 1-pyrroline-5-carboxylic acid (P5C) in the process (Fig. 2). The electrons are then accepted by ubiquinone in the electron transport chain and will then either be used to pump protons (and synthesize ATP) or may leak to generate ROS. Indeed, in some organisms, proline has been demonstrated to be a key source of ATP, which may well indicate why PRODH may be important under some conditions, such as metastasis (Scott et al. 2019; Bergers and Fendt 2021; Elia et al. 2017) or nutrient starvation (Liu et al. 2012). However, over-expression of PRODH has also been shown to elicit apoptosis through ROS generation (Hu et al. 2007; Liu et al. 2009, 2005, 2006, 2008). What determines whether ROS or ATP is generated is unknown, and may be dependent on the nutrient status of the mitochondria—when the ETC is reduced due to a high NADH:NAD^+^ ratio, ROS may be more likely to be generated, while under conditions where nutrients are more limited ATP generation may dominate. Interestingly, during hypoxia and normal nutrient supply, it has been suggested that PRODH overexpression can induce autophagy as an alternative means of generating ATP (Liu et al. 2012). In apparently contradictory evidence, proline supplementation has been suggested to reduce ROS levels at an organismal level. In a rat model of cholestasis/cirrhosis, proline supplementation was able to reduce serum markers of oxidative stress after bile duct ligated (BDL) surgery (Heidari et al. 2018), while in white shrimp proline supplementation was capable of increasing the total antioxidant capacity, PRODH levels and nitric oxide (NO) (Xie et al. 2015). The same result was shown in Angiotensin-II infused rats where proline supplementation decreased urinary H_2_O_2_ while enhancing NO availability, thereby preventing the expected rise in blood pressure (Leal et al. 2019). These apparent contradictions may be resolved through the ability of proline to act as an antioxidant, which may have benefits systemically, while the local effect of proline (through PRODH) may sometimes be pro-oxidative. Interestingly, a model of proline auxotrophy has been recently described in NADK2-deficient tumors. In this system, loss of mitochondrial NADK2, one of the two enzymes which phosphorylates NAD^+^ to NADP^+^, depleted the mitochondrial NADP^+^ pool, reducing ALDH18A1 activity and thereby the ability of cells to synthesize proline (Fig. 3B) (Tran et al. 2021). Importantly, these cells were dependent on exogenous proline and unable to proliferate in proline-starved microenvironments (Tran et al. 2021). The same is observed in fibroblasts from NADK2-deficient patients, which are incapable of sustaining proline biosynthesis and demonstrate NADPH deficiency (Pomerantz et al. 2018). These individuals show increased systemic proline levels, suggesting that systemic proline metabolism can compensate for local and intracellular proline deficiencies, but this is unable to resolve the effects on tissue redox homeostasis.

### Proline cycling

Through this review, the proline synthetic and catabolic pathways utilizing PYCR enzymes and PRODH, respectively, have been considered as linear. However, it is also possible for these pathways to act as part of a proline cycle whose role it is to shuttle proline and its intermediates between the mitochondria and cytosol to move reducing potential between these compartments. Some interesting findings support these interlinked pathways being coordinately regulated, acting as a redox balancing network rather than a cycle per se. One of the most intriguing findings is that lactate, produced as a result of a high cytosolic NADH:NAD^+^ ratio, can inhibit PRODH at physiologically relevant concentrations (Kowaloff et al. 1977). This would likely inhibit this enzyme within hypoxic areas of solid tumors, and the proline cycle might not be active under these conditions.

### Targeting proline metabolism

Over the past decades, a significant body of evidence has shed light on the metabolic regulation of proline availability and consumption, intersecting systemic with local factors. Nevertheless, there are still many open questions, including the role of PYCR3/L in disease or how to effectively and specifically target proline metabolism. Some experimental approaches have trialed the use of inhibitors against PYCR1 or PRODH. Interestingly, no inhibitor for PYCR2 or PYCR3/L has yet been developed, but given the subtle differences in the regulation of these three enzymes, it is likely essential to better understand these to unravel the most relevant targets when treating specific phenotypes. The exploration of the microenvironment and the systemic dynamics have revealed some new targetable subgroups of cells in which proline metabolism is altered during disease. However, specificity may be challenging, with on-target side-effects potentially limiting in terms of a viable therapeutic window. Despite this, with recent evidence promoting the essentiality of proline biosynthesis in CAFs, specific microenvironmental conditions in which PYCR activity may be essential, and the role of PRODH in metastasis, targeting specific areas of cancer biology is very attractive.

## Concluding remarks

Increased proline availability in the environment is the result of local and systemic metabolic integration, and often exerts a protective effect—leveraged in the context of disease to support proliferation and survival. Even if a short exposure to enhanced proline levels seems beneficial, chronic overload or depletion of proline can cause, respectively, toxicity and apoptotic signaling. Yet, the circumstance in which proline becomes a crucial vulnerability is still not clear and must be further explored, not only in the local or systemic environment but also from a temporal perspective. Indeed, proline biosynthetic enzymes might be upregulated even before the detectable disease appears, for instance, during fibrosis and obesity, both of which are known drivers of cancer. Continued metabolic modeling is required to fully understand the underpinning biology of the proline biosynthetic and catabolic machinery, how it responds to different microenvironmental conditions as well as crosstalk between cell types within more complex organ structures. Only then will we be able to predict those situations where pharmacologically targeting enzymes in this pathway may be possible through a potentially tight therapeutic window.

## Data Availability

Not applicable.
